# Are surgical outcomes for one level anterior decompression and fusion associated with MRI parameters for degenerative cervical myelopathy?

**DOI:** 10.3389/fsurg.2022.967269

**Published:** 2022-09-21

**Authors:** Luqiang Qu, Shaofeng Yang, Lijie Yuan, Junjie Niu, Dawei Song, Songping Yang, Huilin Yang, Jun Zou

**Affiliations:** ^1^ Department of Orthopaedic Surgery, The First Affiliated Hospital of Soochow University, Suzhou, China; ^2^ Department of Orthopaedic Surgery, Taicang Affiliated Hospital of Soochow University, Taicang, China; ^3^ Department of Operating Room, The First Affiliated Hospital of Soochow University, Suzhou, China

**Keywords:** DCM, ACDF, MCC, MSCC, CR, RCSCDS

## Abstract

**Background:**

Our study is to determine the correlation between preoperative MRI parameters of spinal cord compression and the effects of anterior surgery in patients with degenerative cervical myelopathy (DCM).

**Methods:**

24 normal subjects with no evident abnormalities were selected as group A. 79 patients with DCM underwent single-segment (C4–5/C5–6) ACDF surgery formed the operation group, and separated into group B (without high signal) and group C (with high signal) according to the absence or presence of high signal in the spinal cord on preoperative T2-weighted MRI respectively. MRI parameters (MCC, maximum canal compromise; MSCC, maximum spinal cord compression; CR, spinal cord compression rate; RCSCDS, ratio of cervical spinal cord to dura sac) were measured. The JOA score was used to evaluate cervical spinal cord function and recovery rate (RR) was used to evaluate postoperative efficacy. The relationship between preoperative MRI parameters and postoperative efficacy was analyzed.

**Results:**

The preoperative JOA score and RR of group B were higher than that of group C. MCC and MSCC in group B were significantly lower than those in groups C. The multiple linear regression equation was the fitted postoperative JOA score = 13.371–2.940 * MCC −5.660 * RCSCDS +0.471 * preoperative JOA score. The fitted RR = 1.451–0.472 * MCC −1.313 * RCSCDS.

**Conclusion:**

The occurrence of high signal on T2-weighted images could reflect more serious spinal cord injury. The postoperative JOA score was significantly correlated with MCC, RCSCDS, and preoperative JOA score, while RR was significantly associated with MCC and RCSCDS.

## Introduction

Degenerative cervical myelopathy (DCM) describes a chronic cervical spine disease characterized by a set of clinical signs and symptoms caused by cervical degeneration and cervical spinal cord compression. Patients with DCM may experience numbness in the limbs, a sense of tightness in the chest, decreased fine motor skills in the hands, a sense of “cotton under the feet” and sphincter dysfunction (1). Diagnosis of DCM mainly rely on clinical evaluation supported by magnetic resonance imaging (MRI). MRI not only reveals anatomical factors of spinal cord compression, but also pathological changes in the spinal canal (2–4). Takahashi first described high signal in the spinal cord on T2-weighted MRI in patients with DCM (5). Some authors subsequently reported that high signal in the spinal cord predicted a worse prognosis after decompression surgery (6). In contrast, others found no correlation between high signal(s) in the spinal cord and postoperative outcomes (7, 8). As such, controversy persists regarding the pathophysiology of spinal cord's T2-weighted signal changes and their relationship with clinical prognosis.

**Figure 1:**
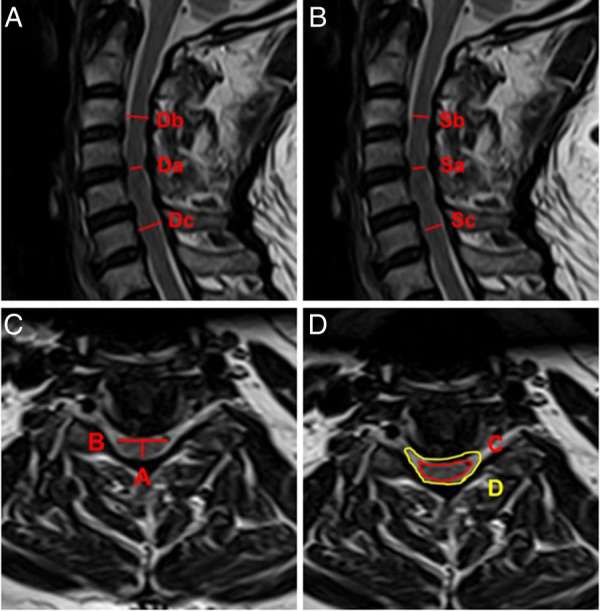
Schematic diagram of spinal cord compression parameter measurement on MRI. (**A**) MCC = [1 − 2Da/(Db + Dc)] × 100%. (**B**) MSCC = [1 − 2Sa/(Sb + Sc)] × 100%. (**C**) CR = A/B × 100%. (**D**) RCSCDS = C/D.

Several attempts have been made to correlate the degree of spinal cord compression on MRI with clinical severity including others’ recent work (9). Quantitative MRI measurements have been, and commonly used measurement parameters include spinal cord cross-sectional area (TA) and spinal cord compression rate (CR) (10, 11). In addition, the ratio of cervical spinal cord to dural sac (RCSCDS), which objectively reflects the relative size of the spinal cord and dural sac during the development of DCM, as well as the degree of spinal cord compression, is a commonly used MRI measurement parameter. Studies have also shown that RCSCDS has important diagnostic and prognostic value in DCM. Okada et al. measured the transverse area of the spinal canal, the dural tube and the spinal cord using MRI in normal adults and patients of DCM and found the ratio of the spinal cord to the spinal canal showed significant correlations with the severity of neurological symptoms. High ratio of the spinal cord to the spinal canal was a responsible static factor for DCM (12). In this study, sagittal measurement parameters, including MCC and MSCC, and transection measurement parameters, including CR and RCSCDS, were used to assess the degree of spinal cord compression (Figure 1).

Surgical strategies for cervical DCM are either anterior, posterior, or combined anterior and posterior approaches. Anterior surgery can either be anterior cervical decompression and fusion (ACDF) or anterior cervical corpectomy and fusion (ACCF). ACDF is considered the gold standard for the management of DCM involving one to two segments, commonly C4–5 or C5–6 (13, 14). Posterior surgery is more suitable for DCM patients with >3 affected segments.

Whether the presence of high signal intensity on T2-weighted MRI in patients of DCM indicated worse prognosis is full of controversy. Chi-Jen Chen et al. found when the high signal intensity was predominantly faint with a fuzzy border there was no significant difference in prognosis. Whilst, when the high signal intensity was predominantly intense and well-defined border the prognosis became worse (15). However, Wada believed that high intensity areas on T2-weighted MRI were not correlated with the severity or surgical outcomes of DCM (8). More studies need to be undertaken to investigate the relationships between MRI indicators and prognosis or surgical outcome in patients of DCM.

The purpose of this study was to investigate the correlation between preoperative MRI indicators reflecting spinal cord compression (i.e., MCC, MSCC, CR, and RCSCDS) and the efficacy of anterior surgery in patients with DCM with the goal of providing some imaging references for the prognosis of DCM.

## Materials and methods

The clinical study was approved by the Ethics Committee of the authors’ affiliated institutions and written informed consents were obtained from all participants. This is a prospective uncontrolled non-randomized study performed pragmatically where patients having undergone 1 level ACDF for DCM were separated based on cord signal changes and then compared to a cohort of healthy volunteers for MRI findings. The control group (group A) consisted of 24 subjects [10 male, 14 female; mean (±SD) age 49.5 ± 6.21 years] who underwent MRI of the cervical spine in the outpatient department and exhibited no obvious abnormalities or surgical indications (Table 1). From January 2017 to December 2018, 79 patients with DCM underwent single-segment (C4–5/C5–6) ACDF were selected as the operation group, which was subdivided into group B (without high signal) and group C (with high signal) according to the presence or absence of high signal in the spinal cord on T2-weighted images on preoperative MRI. There were 52 patients in group B, including 30 males and 22 females, with an average age of 54.1 ± 5.23 years. A total of 29 patients underwent ACDF at C4–5 and 23 underwent ACDF at C5–6 levels. There were 27 patients in group C, including 17 males and 10 females, with an average age of 59.3 ± 3.89 years. A total of 15 patients underwent ACDF at C4–5 and 12 underwent ACDF at C5–6; all patients underwent preoperative MRI examination. The inclusion criteria for the operation group (groups B and C) were signs and symptoms of DCM; MRI revealing spinal cord compression; underwent anterior cervical surgery at one level between C4 and C6; and had no history of cervical spine surgery. Individuals with other types of cervical spondylosis, such as nerve root compression, sympathetic symptoms, esophageal and vertebral artery pathology, those with ankylosing spondylitis, a history of cervical spine trauma, rheumatoid arthritis, cervical tuberculosis, tumor(s), amyotrophic lateral sclerosis, ACDF surgery for ≥2 segments, and those who underwent ACCF or non-C4 to C6 single-segment ACDF surgery, were excluded.

**Table 1:** Comparison of baseline characteristics of patients between three groups.

Index	Group A	Group B	Group C
Age (Y)	49.5 ± 6.21	54.1 ± 5.23	59.3 ± 3.89
Male (N)	10	30	17
Female (N)	14	22	10
Segment (C4–5)	15	29	15
Segment (C5–6)	9	23	12
Total (N)	24	52	27

Preoperative MRI of the cervical spine was performed in all patients, and parameters were measured at the most severe level of spinal cord compression in the sagittal position and the transverse position using T2-weighted imaging. The main parameters measured in the sagittal position were maximum canal compression (MCC) and maximum spinal cord compression (MSCC). The main parameters measured in the transverse position were CR and RCSCDS. The measurement methods for each parameter were as follows: MCC = [1 − 2Da/(Db + Dc)] × 100%, in which Da, Db, and Dc represent the sagittal diameter of the spinal canal in the stenotic segment, the sagittal diameter of the spinal canal in the segment above the stenotic segment, and the sagittal diameter of spinal canal in the segment below the stenotic segment, respectively (Figure 1A); MSCC = [1 − 2Sa/(Sb + Sc)] × 100%, in which Sa, Sb, and Sc, represent the sagittal diameter of the spinal cord in the stenotic segment, the sagittal diameter of the spinal cord in the segment above the stenotic segment, and the sagittal diameter of the spinal cord in the segment below the stenotic segment, respectively (Figure 1B); CR = A/B × 100%, in which A and B represent the minimum vector diameter and maximum transverse diameter of the compressed part of the spinal cord, respectively (Figure 1C); and, finally, RCSCDS = C/D, in which C and D, represent the area of the spinal cord and the area of the dural sac at the transverse position of spinal cord compression, respectively (Figure 1D). All measurements were independently recorded by two orthopedic surgeons; each indicator was measured three times by each surgeon and the mean value was calculated and used in the analysis.

The evaluation criteria of spinal cord function developed by the Japanese Orthopedic Association (JOA) were used. The postoperative recovery rate (RR) was used to evaluate the effect of surgery according to the following equation:


$$\begin{array}{r} \mathrm{RR}=\;\left(\;\mathrm{postoperative}\;\mathrm{JOA}\;\mathrm{score}\;-\mathrm{preoperative}\;\mathrm{JOA}\;\mathrm{score}\right)/\\ \left(17\;-\mathrm{preoperative}\;\mathrm{JOA}\;\mathrm{score}\right)\times 100\% \end{array}$$

Sigma plot version 14 (Systat Software Inc, San Jose, CA, USA) was used to analyze the data, which are expressed as mean ± standard deviation, and comparison among groups was performed using one way analysis of variance (ANOVA). Pearson correlation analysis was used to analyze the correlation between MRI parameters, JOA score, and RR. When the absolute value of the correlation coefficient is greater than 0.7, it is defined as high correlation, when it is between 0.4 and 0.7, it is defined as moderate correlation, and when it is less than 0.4, it is defined as mild correlation. Multiple linear regression analysis was used to obtain the fitted postoperative JOA score and RR, and Pearson correlation coefficient was used to test the correlation between the indexes. Differences with *P* < 0.05 were considered to be statistically significant.

## Results

**Figure 2:**
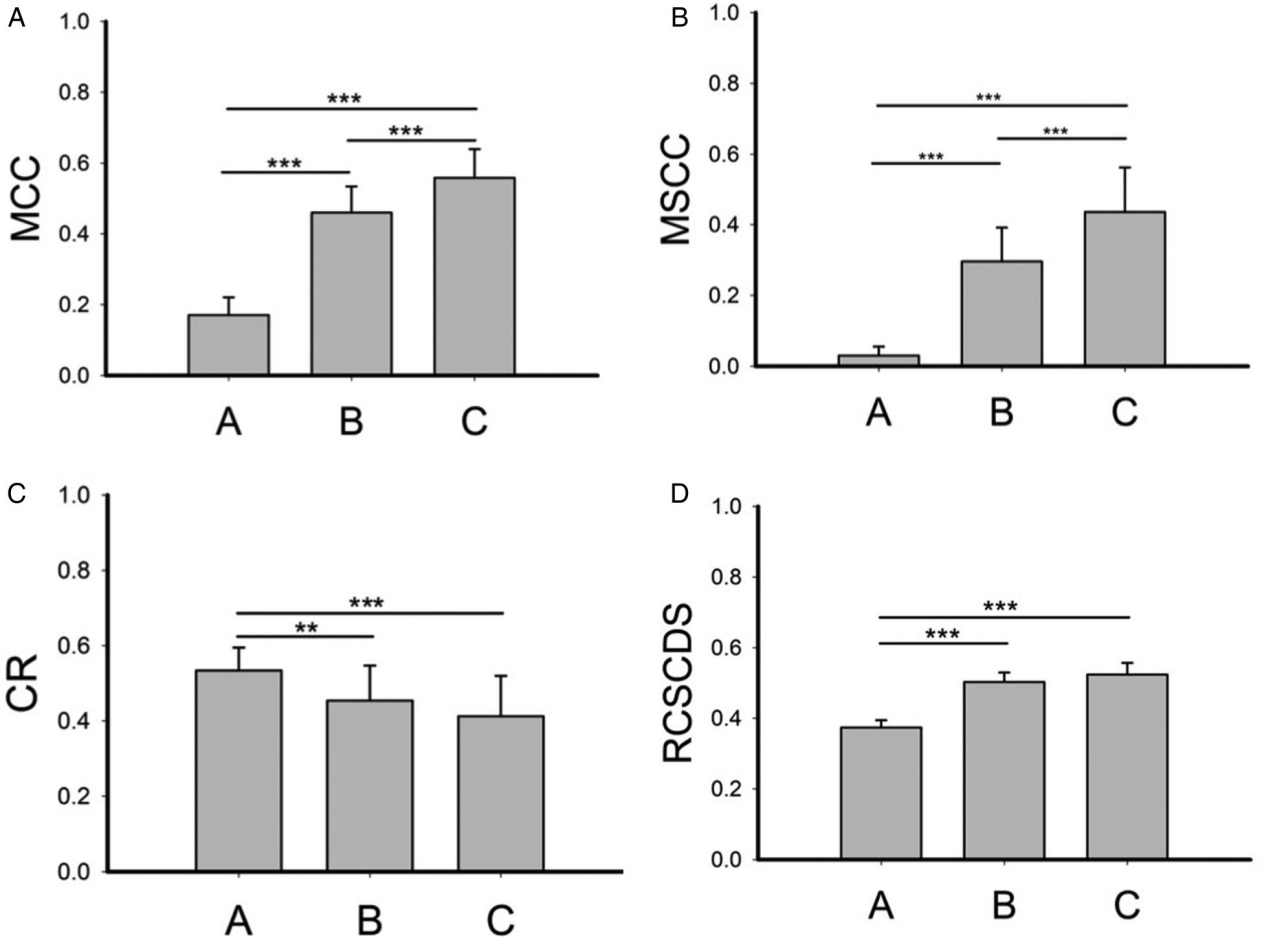
The spinal cord compression parameters of each group. MCC, maximum canal compromise; MSCC, maximum spinal cord compression; CR, compression ratio; RCSCDS, ratio of cervical spinal cord to dura sac. **P < 0.01; ***P < 0.001.

The mean MCC (Figure 2A), MSCC (Figure 2B), CR (Figure 2C) and RCSCDS (Figure 2D) in group A, B, and C were calculated in Figure 2. The four MRI parameters of spinal cord compression in the operation group were larger than those in the control group, while in the operation group, when there was high signal in the spinal cord, the MCC and MSCC increased.

**Figure 3:**
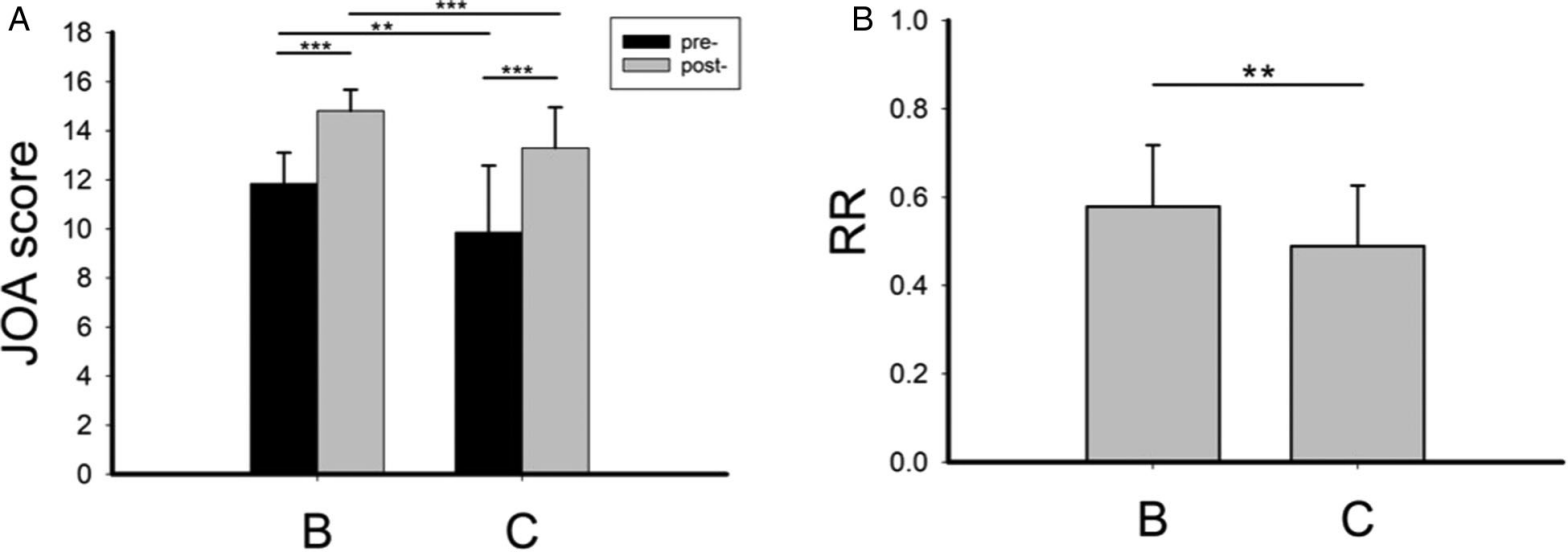
The preoperative and postoperative JOA score (**A**) and postoperative recovery rate (**B**) in group B and group C. RR, recovery rate. **P < 0.01; ***P < 0.001.

As shown in Figure 3, in the operation group, preoperative JOA score, postoperative JOA score (Figure 3A), and RR (Figure 3B) were significantly reduced when high signal in the spinal cord was present (group C), indicating more severe DCM and worse postoperative efficacy when a high signal was present in the spinal cord.

**Figure 4:**
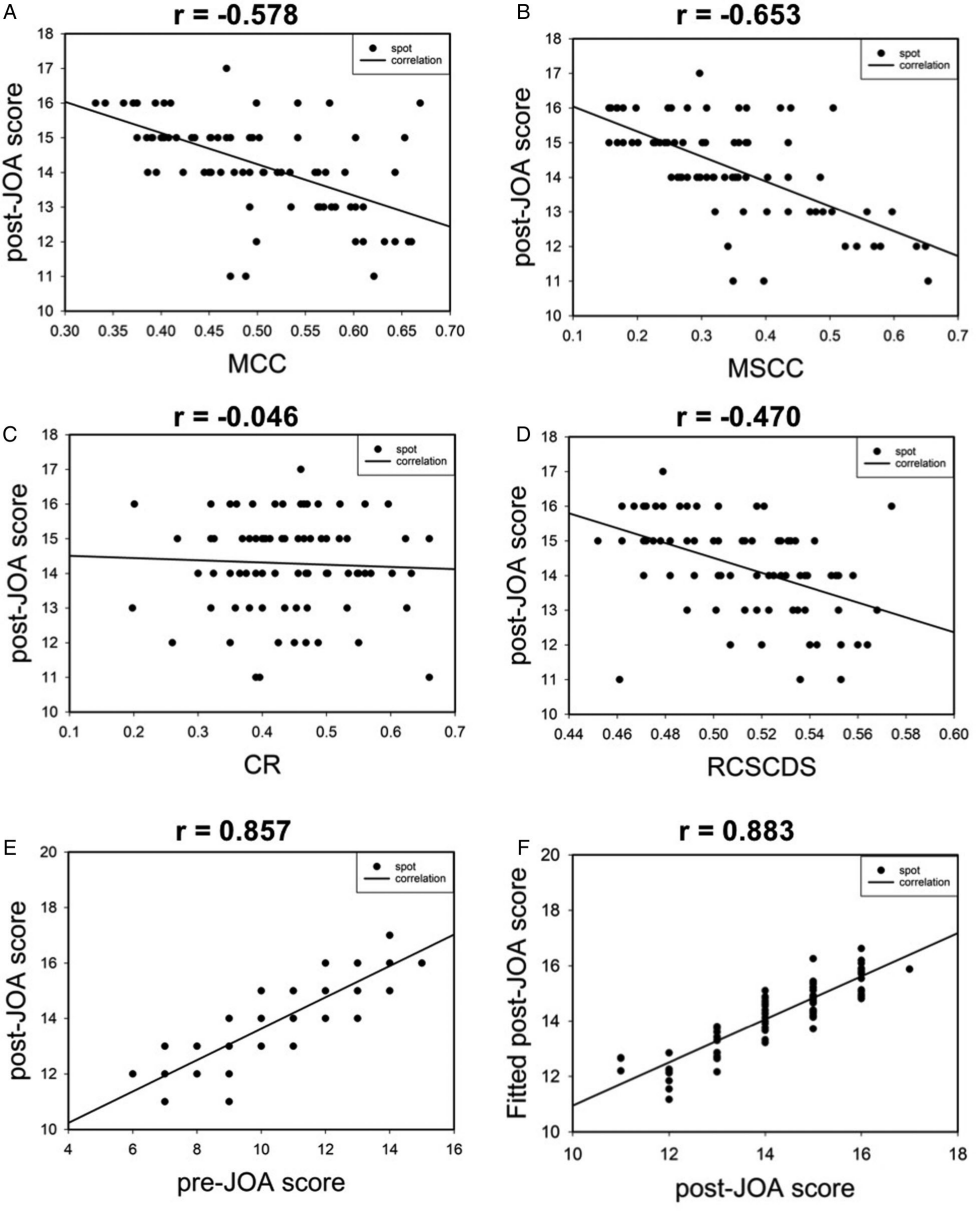
The scatter plot of correlation between postoperative JOA score and preoperative spinal cord compression parameters (**A–D**). Correlation between postoperative JOA score and preoperative JOA score (**E**). Correlation between postoperative JOA score and fitted postoperative JOA score (**F**).

The correlation between postoperative JOA score and MRI parameters of spinal cord compression preoperative JOA score were analyzed. Results revealed that there were moderate, moderate, not correlated, moderate, and high correlation between postoperative JOA score and MCC, MSCC, CR, RCSCDS, and preoperative JOA score, respectively. In addition, the results of multiple linear regression analysis are shown in Figure 4, and the equation was as follows: fitted postoperative JOA score = 13.371 – 2.940 * MCC − 5.660 * RCSCDS + 0.471 * preoperative JOA score. As shown in Figure 4F, the actual postoperative JOA score was highly correlated with the fitted postoperative JOA score, with a correlation coefficient of 0.883 (*P* < 0.05).

**Figure 5:**
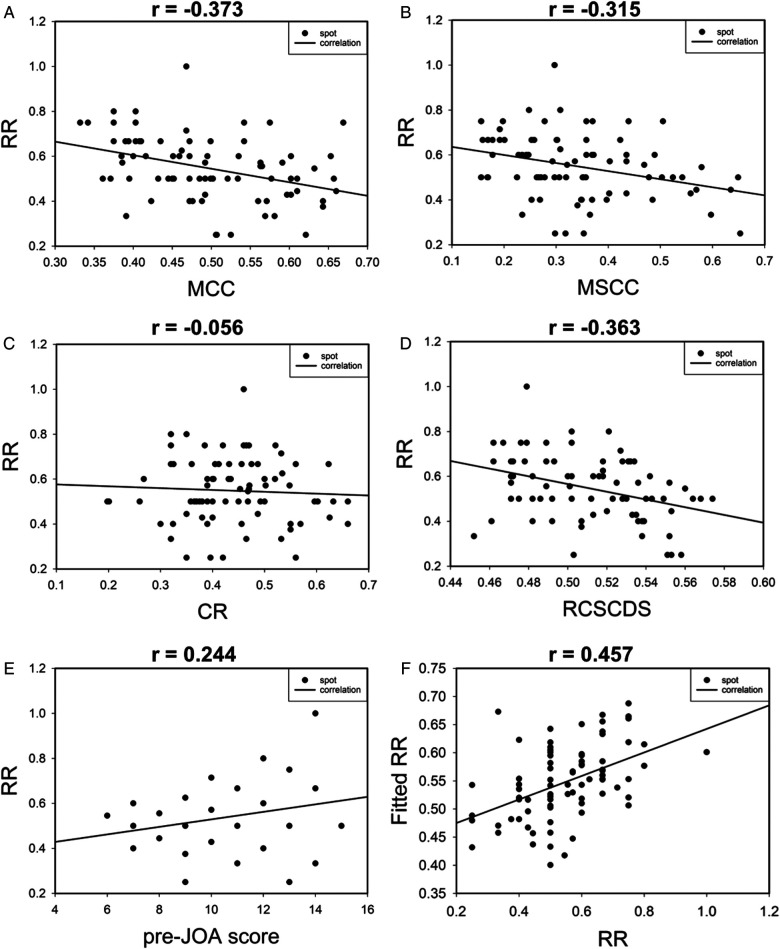
The scatter plot of correlation between postoperative recovery rate and preoperative spinal cord compression parameters (**A–D**). Correlation between postoperative recovery rate and preoperative JOA score (**E**). Correlation between postoperative recovery rate and fitted postoperative recovery rate (**F**).

The correlation between RR and MRI parameters of spinal cord compression and preoperative JOA score was analyzed. The results revealed moderate, moderate, not correlated, moderate, and moderate correlations between RR and MCC, MSCC, CR, RCSCDS, and preoperative JOA score, respectively. In addition, the results of multiple linear regression analysis are shown in Figure 5, and the equation was as follows: fitted RR = 1.451–0.472 * MCC −1.313 * RCSCDS. The actual RR was highly correlated with the fitted RR, with a correlation coefficient of 0.457 (*P* < 0.05) (Figure 5F).

## Discussion

DCM occurs mainly due to the degeneration of cervical region structures in the spine, causing spinal stenosis and spinal cord compression, resulting in a series of clinical symptoms. There are several measures available to determine the degree of spinal cord compression. Fehlings et al. used MCC, MSCC, CR, and TA to reflect the degree of spinal cord compression (16). The space between the cervical spinal canal and the cervical spinal cord reflects the compensatory ability of DCM patients when the cervical spinal cord is compressed. The RCSCDS reflects the relative size of the spinal cord and dural sac in the process of DCM development. Compared to TA (i.e., cross-sectional area of the spinal cord), RCSCDS better reflects the degree of spinal cord compression; therefore, we selected RCSCDS as one of the measurement indicators in this study.

Many studies have investigated predictors of surgical outcome(s) for DCM. Okada et al. believed that the postoperative outcome of DCM was significantly related to the cross-sectional area of the most severely affected segment of spinal cord compression, disease course, and high signal intensity in the spinal cord (10). In addition, Jinkins et al. found that the cross-sectional area of the most severely affected segment of spinal cord compression was related to signal intensity in the spinal cord (3, 17). We found that factors influencing postoperative JOA score included MCC, RCSCDS, and preoperative JOA score, excluding MSCC and CR. Furthermore, Nouri and Tetraault et al. found a significant correlation between post- and preoperative spinal cord function in those with DCM (18, 19). In addition, the coefficient of MCC and RCSCDS in the regression analysis was negative, and the coefficient of preoperative JOA score was positive. This indicates that the larger the MCC and RCSCDS, the smaller the reserve space in the direction of sagittal and transverse position of the spinal cord, and the lower the functional score in the spinal cord, the worse the function of the spinal cord. Higher preoperative JOA score was associated with better postoperative spinal cord function. The factors affecting RR included MCC and RCSCDS. In addition, the coefficient of MCC and RCSCDS in the regression analysis was negative, indicating that the greater the MCC and RCSCDS, the worse the postoperative efficacy.

The present study has several shortcomings and limitations. First, the retrospective design led to an inherent bias, which, together with the relatively small number of cases, may have made the results prone to error. Furthermore, the JOA score and MRI parameter measurements were manually scored and processed using Picture Archiving and Communication software, which is prone to measurement deviation. Second, the JOA score and RR may be affected by many other factors, including age, disease course, high signal intensity in the spinal cord, and operation time. Although we divided the operation group into groups B and C, we did not analyze high signal in the spinal cord as an influencing factor.

## Conclusion

In conclusion, MCC, MSCC, CR, and RCSCDS reflected spinal cord compression on MRI and preliminarily suggested whether there is an objective indication for surgery. The occurrence of high signal in the spinal cord on T2-weighted images could reflect more serious spinal cord injury, and also suggested that early intervention should be performed before the occurrence of high signal in DCM. MCC and MSCC could, to some extent, reflect the severity of spinal cord compression. JOA score was significantly correlated with MCC, RCSCDS, and preoperative JOA score. The RR was significantly related to MCC and RCSCDS.

## Data Availability

The raw data supporting the conclusions of this article will be made available by the authors, without undue reservation.
